# Peptide Mass Fingerprinting and N-Terminal Amino Acid Sequencing of Glycosylated Cysteine Protease of *Euphorbia nivulia* Buch.-Ham.

**DOI:** 10.1155/2013/569527

**Published:** 2013-02-17

**Authors:** Shamkant B. Badgujar, Raghunath T. Mahajan

**Affiliations:** ^1^Department of Biotechnology, Faculty of Science, Post Graduate College of Science, Technology and Research, North Maharashtra University, Jalgaon, Maharashtra 425002, India; ^2^Department of Biotechnology, Faculty of Science, Moolji Jaitha College, Jalgaon, Maharashtra 425002, India

## Abstract

A new cysteine protease named Nivulian-II has been purified from the latex of *Euphorbia nivulia* Buch.-Ham. The apparent molecular mass of Nivulian-II is 43670.846 Da (MALDI TOF/MS). Peptide mass fingerprint analysis revealed peptide matches to Maturase K (Q52ZV1_9MAGN) of *Banksia quercifolia*. The N-terminal sequence (DFPPNTCCCICC) showed partial homology with those of other cysteine proteinases of biological origin. This is the first paper to characterize a Nivulian-II of *E. nivulia* latex with respect to amino acid sequencing.

## 1. Introduction

Proteases are enzymes which potentially hydrolyze anything that contains peptide bond, from a dipeptide up to a large protein, containing thousands of amino acids and, thus, it comprises a group of hydrolases that are the most relevant in technological terms. Proteolytic enzymes have been the subject of intensive studies for a number of years because proteases have great therapeutic and industrial applications as well [1]. Peptidases in which the nucleophile that attacks the scissile peptide bond is the sulfhydryl group of a cysteine residue are known as cysteine-type peptidase. Many plant proteases have been isolated from the lattices and most of them belong to this catalytic type. Plant cysteine proteases belong to a class which has been widely studied over the years. These enzymes are also used in industries owing to their high stability [2]. Plant cysteine proteases play a major role in the intracellular and extracellular process such as development and ripening of fruits, degradation of storage proteins in germinating seeds, activation of proteins, and degradation of defective proteins [3]. Besides, latex proteases are also involved in the protection of the plants against predator attack [4]. Many researchers have isolated and characterized the plant origin proteases of diverse group.

*Euphorbia nivulia* Buch.-Ham. is a wild, thorny, xerophytic, succulent plant, found in boundaries of the agricultural field and also in dry barren areas. The secretion of milky juice is a characteristic property of this plant. Phytochemical studies have led to the isolation of ingol diterpenes (3-acetyl-8-methoxyl-7-angolyl-12-hydroxylingol; 3,12-diacetyl-7-hydroxy-8-methoxylingol; 3,12-diacetyl-7-angolyl-8-hydroxylingol; 3,12-diacetyl-8-benzoylingol; and 3,12-diacetyl-7-benzoyl-8-nicotinylingol) along with three macrocyclic ingol diterpenes derivatives (3,7,12-triacetyl-8-benzoylingol; 3,12-diacetyl-7-angeloyl-8-methoxyingol; and 7-angeloyl-12-acetyl-8-methoxyingol) [5]. The latex of *E. nivulia* has been cited for its antioxidant, immunomodulator, cytotoxic, anti-inflammatory, wound healing, haemostatic, and antiproliferative activity [6]. During the course of screening for biochemical constituents, a substantial amount of proteolytic and milk clotting activity was found in the latex of this plant [7]. Recently, an attempt has been made on peptide sequencing of 31-kDa, Tubulin alpha-1 chain-like protein called nivulian-I, present in the latex of *E. nivulia* [8]. Very recently, a comparative account on proteolytic activity of *E. nivulia* and other three plants, namely, *Calotropis procera*, *Carica papaya*, and *Ficus carica*, was reported by us. Additionally, we characterized the glycosylated cysteine protease called Nivulian-II of the latex of *E. nivulia* [5]. This paper describes the biochemical characterization, of this cysteine like protease with respect to peptide fingerprinting and N-terminal amino acid sequencing.

## 2. Materials and Methods

### 2.1. Chemicals

Sigma Chemicals: phenyl isothiocyanate (PITC), N-methylpiperidine, Methanol, Trifluoroacetic acid, Dithiothreitol, Acetonitrile, 20 Amino acid PTH standards, etc. were thankfully gifted by the Department of Biochemistry, Indian Institute of Science, Bangalore (Karnataka), India. Sequencing grade Hybond-PVDF membrane (Amershyam) was gifted by the Department of Biochemistry, TMC, Advanced centre for Treatment, Research and Education in Cancer (ACTREC), Navi Mumbai, India. All other chemicals were of the highest purity, analytical HPLC grade purchased from Himedia Laboratories, Mumbai; SRL Chemicals, Bangalore; Qualigen Fine Chemicals, Mumbai; Merck Chemicals, India; and Bangalore Genie, India.

### 2.2. Plant Material, Collection of Latex, Crude Enzyme Preparation, and Purification of Cysteine Protease

The detail information about identification, collection, preservation, preparation of crude enzyme and its proteolytic activity of *Euphorbia nivulia* latex is described in our previous communication [7]. Method of purification of protease was done using acetone precipitation, DEAE cellulose chromatography, dialysis and followed by rechromatography on DEAE cellulose column as described in our earlier communication [5]. 

### 2.3. Mass Spectroscopy

Matrix-assisted laser desorption ionization/time of flight mass spectroscopy (MALDI/TOF MS) was used for the determination of the molecular mass, as well as the degree of purity of active enzyme. MALDI/TOF mass spectra were acquired on a Bruker Daltoncs model Ultraflex II Spectrometer, Germany, equipped with a pulsed Neodymium yttrium-aluminium garnet (Nd-YAG) smart beam solid state laser (337 nm), in reflectron positive-ion mode, using a 19-kV acceleration voltage. Enzyme samples were prepared by mixing equal volumes of a saturated solution of the matrix that is, sinapinic acid (prepared in 0.1% TFA (aq.) and 50% acetonitrile (2 : 1)). 2 *μ*L matrix solution and 2 *μ*L protein (enzyme) fraction were spotted on the sample plate, mixed them, and allowed to evaporate to dryness. Proteins of known molecular mass (insulin, ubiquitin I, cytochrome C, and myoglobin) were used as standards for mass calibration.

### 2.4. Peptide Analysis

For peptide mass fingerprinting, the targeted protein band (spot) was manually excised from the gel and was processed for MS and MS/MS analysis as described previously [9]. In brief, gel pieces were washed twice with 50% (v/v) acetonitrile in 50 mM ammonium bicarbonate, shrunk by dehydration in acetonitrile and dried in vacuum centrifuge. Disulfide bonds were reduced by incubation with 10 mM dithiothreitol (DTT) in 50 mM ammonium bicarbonate for 45 min at 56°C. Alkylation was performed by replacing the DTT solution with 55 mM iodoacetamide in 50 mM ammonium bicarbonate. After 30 min incubation at 25°C in the dark, the gel pieces were washed with 50% (v/v) acetonitrile in 50 mM ammonium bicarbonate, shrunk by dehydration in acetonitrile, and dried in vacuum centrifuge. The dried gel pieces were incubated with sequencing-grade modified trypsin at 37°C overnight. To extract the tryptic digested peptides, 0.1% trifluoroacetic acid in acetonitrile was added, the samples were sonicated for 5 min and the separated supernatant was dried under vacuum. Then, the samples (peptides) were desalted using a Ziptip C 18 (Millipore, USA) according to the manufacturer's protocol. MALDI-MS measurements were performed using MALDI TOF/MS in reflectron mode using *α*-cyano-4-hydroxycinnamic acid as a matrix. All mass spectra were internally calibrated with trypsin autolysis peaks. Peptide mass fingerprinting (PMF) was carried out using MASCOT Matrix Sciences (London, UK) program for protein identification (http://www.matrixscience.com/). MALDI-TOF/TOF fragment ion analysis (see Figure  S1 in supplementary Material available online at http://dx.doi.org/10.1155/2013/569527) of selected individual parent peptide (Figure  S2) was carried out in the LIFT mode of the instrument. Then, in order to further confirm the identification, all MS/MS data from LIFT TOF/TOF spectra were combined with the corresponding MS peptide mass fingerprinting data for database searching.

The spectra were analyzed with flex-Analysis software (Version 3.2, Bruker-Daltonics, Germany) and searched against two taxonomies: (i) other green plants and (ii) species information unavailable in the NCBI (NCBInr) and MSDB database using MASCOT software (Version 2.2). The PMF search parameters were: 100 ppm tolerance as the maximum mass error, MH^+^ monoisotopic mass values, allowance of oxidation (M) modifications, allowed for 1 missed cleavage, and fixed modification of cysteine by carboxymethyl, that is, Carbamidomethylation (C) [10].

### 2.5. N-Terminal Amino Acid Sequence Analysis

The purified protease enzyme was adsorbed onto a PVDF membrane and washed several times with deionised water. The N-terminal sequence was determined by Pehr Edman's automated degradation method using a Procise protein sequencing system (Applied Biosystem) composed of four integrated modules. This system sequentially cleaves N-terminal amino acids from protein and analyzes the resulting phenylthiohydantoin (PTH) amino acid residues. Protein homology searches were performed using the BLAST [11], indicating the specific residues which are identical “identities” as well as those which are nonidentical, but nevertheless have positive alignment scores “Positives”. 

## 3. Results and Discussion

*Euphorbia nivulia* Buch.-Ham. belongs to the Euphorbiaceae family, whose members are characterized by secretory tissues (laticifers) which frequently include proteolytic and milk clotting enzymes. The young stem latex of *E. nivulia* possesses proteolytic and milk clotting enzyme in more quantity as compared with other investigated laticiferous plants of Northern region of Maharashtra, India [5]. *E. nivulia* latex contains thermostable glycosylated cysteine protease having 6.6 and 45°C are, respectively, optimum pH and temperature, molecular weight is about 43.42 kDa (SDS-PAGE), cysteine hydrochloride activator, and inhibitor is mercuric chloride [5]. According to protease nomenclature, this protein is designated as Nivulian-II due to previously Nivulian-I (31486.985 Da) was already characterized from *E. nivulia* latex [8]. 

**Figure 1:**
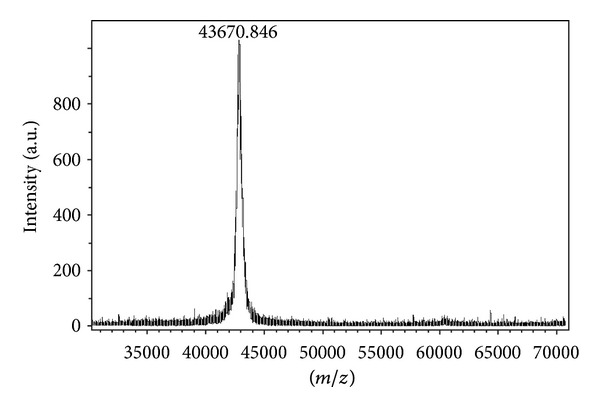
Mass spectroscopy of Nivulian-II of *Euphorbia nivulia* latex.

Mass spectroscopy (Figure 1) revealed that Nivulian-II had a molecular mass of 43670.846 Dalton (43.67 kDa), which is relatively matched with molecular mass determined by SDS-PAGE, that is, 43.42 kDa [5, 12]. Similar molecular pattern was also recorded in the molecular mass measurement of Funastrain CII [13]. The resulted molecular mass, that is, 43.67 kDa is very close to those of other peptidase belonging to cysteine protease family for example cysteine proteases of *Vigna mungo*, *Curcuma longa*, *Taenia crassiceps*, *Allomyces aebuscula*, *Triticum arstivum*, *Haemonchus contortus*, and *Gymnophalloides seoi* [12]. 

**Figure 2:**
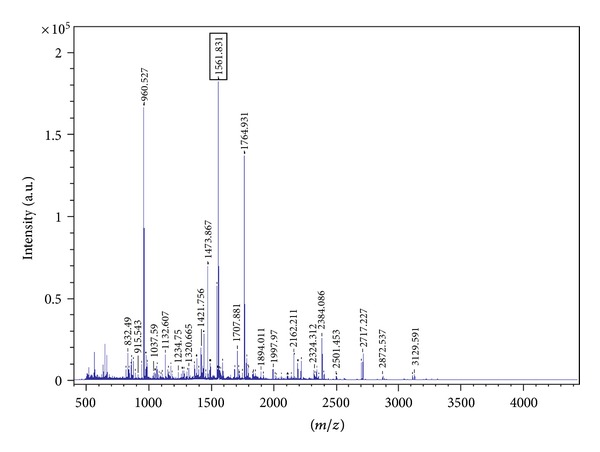
MALDI-TOF mass spectrum of trypsin-digested peptide map of Nivulian-II.

**Figure 3:**
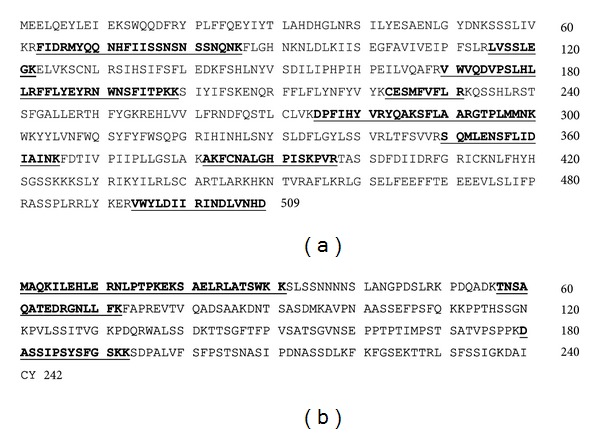
(a) Peptides of Nivulian-II matched with Maturase K (Q52ZV1_9MAGN) of *Banksia quercifolia.* (b) Peptides of Nivulian-II matched with unknown protein (gi*|*118485477) of *Populus trichocarpa. *

Out of total trypsin-digested peptide fragments (Figure 2) of Nivulian II, only thirteen peptides that is, 28.50% sequence was hit in MSDB (mass spectrometry protein sequencing database) by Mascot peptide fingerprint search engine with Maturase K (Q52ZV1_9MAGN) of *Banksia quercifolia* (Family: Proteaceae) with 67.1 score and *P* < 0.05 (Figure 3(a)). An additional match of the mascot peptide mass fingerprint of Nivulian-II with other existing protein sequence from NCBI (National Center for Biotechnology Information) database was also performed. One of the unknown protein (gi*|*118485477) of *Populus trichocarpa* (Family: Salicaceae), matches 7 peptides (i.e., 25.2% sequence) of Nivulian-II as shown in Figure 3(b) (http://www.matrixscience.com/).

The mass spectrum showed several protonated ions [M+H]^+^ of the peptide fragments. As listed in Table 1, the ions at 832.490, 1037.590, 1046.635, 1154.597, 1188.631, 1234.750, 1552.775, 1561.831, 1794.938, 1852.035, 1997.970, 2357.131, and 2872.537 were the thirteen trypsin digested peptides corresponding to residues 115–122, 183–189, 275–282, 283–292, 223–231, 190–199, 287–300, 170–182, 382–397, 350–365, 494–509, 67–86 and 63–86. These were designated as N2T1, N2T2, N2T3, N2T4, N2T5, N2T6, N2T7, N2T8, N2T9, N2T10, N2T11, N2T12, and N2T13, respectively. As depicted in Table 1, peptide mass profiles were obtained from the database search engine and amino acid sequence of individual peptides were identified from known sequence of Maturase K of *Banksia quercifolia* from desired spot of Nivulian-II protein of SDS-PAGE.  Table 2 summarizes the amino acid composition of obtained peptide fragments (N2T1 to N2T13) of Nivulian-II by trypsin digestion. Only two residues of OH-group and S-containing amino acid, that is, threonine (T) and cysteine (C), were recorded in sequenced peptides. A total of 171 amino acids were sequenced from Nivulian-II protein. The content of serine (11.65%) and asparagine (10.52%) was abundant in sequenced peptides of Nivulian-II. 

The N-terminal amino acid sequence of cysteine protease named as Nivulian-II was deposited in the universal protein resource database, “Uniprot” under the accession number P86837. A comparative study of homology of N-terminal sequences of various cysteine proteases with P86837 using BLAST algorithm network services (http://blast.ncbi.nlm.nih.gov/Blast.cgi) was conducted. The N-terminal sequence of Nivulian-II was aligned with those of other plant cysteine proteases and showed 25–50% identity with other cysteine peptidases (Tables 3 and 4). The isoleucine (I) is present on the 10th position from N-terminal side of Nivulian-II, which is fully homologous with isoleucine of Maturase K, as it is present on the 10th position from amino terminal side (Figure 3(a)). 

N-terminal sequence of Nivulian-II shows only 25% homology sequence with plant origin cysteine proteases, that is, GP-I and GP-II of *Zingiber officinale* rhizome [14]. It shows 16.66% similarity with bromelain of pineapple stem protease [15]. It is also homologous with some animal origin cysteine protease, that is, cathepsin F [16] and cathepsin J [17] and bacterial cysteine protease, that is, IdeS of *Streptococcus pyogenes *[18]. This sequence showed higher identity (50%) with other cysteine endopeptidase isolated from the latex of rubber tree, *Hevea brasiliensis* Muell, Arg [19], and a member of the Euphorbiaceae family, that is, *Ricinus communis*. It is also homologous (41.66%) with some microbial origin cysteine proteases of *Naegleria gruberi* [20] and *Polysphondylium pallidum*, sulphur-containing protease, that is, Methionine protease (MAP) of *Mycoplasma hominis* and blastula-like protease (BP-10) of *Saccoglossus kowalevskii*. Nivulian-II shared the motifs surrounding the catalytic cysteine (NT and CCI) that also occurred in most of the sequences compared (Table 4). The asparagine (N) is notably conserved and it is present in all the cases as well as the cysteine residue located in position 8.

In the light of all these experimental observations it is envisaged that the present cysteine protease, Nivulian-II, is characterized for the first time. N-terminal amino acid sequence and tryptic digestion profile of nivulian infer the exclusive nature of enzyme and it may be a novel protease of the cysteine family.

## Supplementary Material

Figure S1: Prime peptide (1561.831) of Nivulian-II for MALDI-TOF/TOF fragmention analysis. Figure S2: MALDI-TOF/TOF fragment ion analysis of prime peptide of Nivulian-II (1561.831).

## Figures and Tables

**Table 1:** Calculated and observed ions of trypsin digests of Nivulian-II.

Peak number	Intensity	Amino acid sequence		Sequence	[M+H]^+^	
		From	To		Calculated	Observed
					*m*/*z *	
02	13226.59	115	122	LVSSLEGK	832.477	832.490
18	3770.272	183	189	FFLYEYR	1037.509	1037.590
19	3540.355	275	282	DPFIHYVR	1046.542	1046.635
27	5762.630	283	292	YQAKSFLAAR	1154.632	1154.597
30	2878.922	223	231	CESMFVFLR	1188.554	1188.631
31	3792.376	190	199	NWNSFITPKK	1234.658	1234.750
52	5565.563	287	300	SFLAARGTPLMMNK	1552.797	1552.775
54	71959.36	170	182	VWVQDVPSLHLLR	1561.885	1561.831
67	3587.283	382	397	AKFCNALGHPISKPVR	1794.980	1794.938
70	3325.145	350	365	SQMLENSFLIDIAINK	1851.952	1852.035
73	5424.503	494	509	VWYLDIIRINDLVNHD	1998.044	1997.970
90	1525.765	67	86	MYQQNHFIISSNSNSSNQNK	2357.057	2357.131
98	1297.007	63	86	FIDRMYQQNHFIISSNSNSSNQNK	2872.343	2872.537

**Table 2:** Amino acid composition of peptides obtained from Nivulian-II by trypsin digestion.

Amino acid	Number of amino acids per peptide													Total
	A	B	C	D	E	F	G	H	I	J	K	L	M
Gly (G)	1	—	—	—	—	—	1	—	1	—	—	—	—	03
Ala (A)	—	—	—	3	—	—	2	—	2	1	—	—	—	08
Val (V)	1	—	1	—	1	—	—	3	1	—	2	—	—	09
Leu (L)	2	1	—	1	1	—	2	3	1	2	2	—	—	15
Ile (I)	—	—	1	—	—	1	—	—	1	3	3	2	3	14
Ser (S)	2	—	—	1	1	1	1	1	1	2	—	5	5	20
Thr (T)	—	—	—	—	—	1	1	—	—	—	—	—	—	02
Cys (C)	—	—	—	—	1	—	—	—	1	—	—	—	—	02
Met (M)	—	—	—	—	1	—	2	—	—	1	—	1	1	06
Asp (D)	—	—	1	—	—	—	—	1	—	1	3	—	1	07
Asn (N)	—	—	—	—	—	2	1	—	1	2	2	5	5	18
Glu (E)	1	1	—	—	1	—	—	—	—	1	—	—	—	04
Gln (Q)	—	—	—	1	—	—	—	1	—	1	—	3	3	09
Phe (F)	—	2	1	1	2	1	1	—	1	1	—	1	2	13
Tyr (Y)	—	2	1	1	—	—	—	—	—	—	1	1	1	07
Try (W)	—	—	—	—	—	1	—	1	—	—	1	—	—	03
Lys (K)	1	—	—	1	—	2	1	—	2	1	—	1	1	10
Arg (R)	—	1	1	1	1	—	1	1	1	—	1	—	1	09
His (H)	—	—	1	—	—	—	—	1	1	—	1	1	1	06
Pro (P)	—	—	1	—	—	1	1	1	2	—	—	—	—	06

Total	08	07	08	10	09	10	14	13	16	16	16	20	24	171

Peak number	02	18	19	27	30	31	52	54	67	70	73	90	98	—

A: N2T1; B: N2T2; C: N2T3; D: N2T4; E: N2T5; F: N2T6; G: N2T7;

H: N2T8; I: N2T9; J: N2T10; K: N2T11; L: N2T12 and M: N2T13 Peptides.

**Table 3:** Comparison of N-terminal sequences of Nivulian-II with other known cysteine proteases.

Source	Enzyme	Sequence*												Identities	Positives
*Euphorbia nivulia *	Nivulian-II	**D**	**F**	**P**	**P**	**N**	**T**	**C**	**C**	**C**	**I**	**C**	**C**	—	—
*Opisthorchis viverrini *	Cathepsin F	M	R	**P**	F	V	C	**C**	V	L	**V**	T	T	2/12 (16.66)	3/12 (25.00)
*Streptococcus pyogenenes *	IdeS	K	S	C	D	K	**T**	H	T	**C**	P	P	**C**	3/12 (25.00)	3/12 (25.00)
*Zingiber officinale *	GP-I	**D**	V	L	**P**	D	**S**	I	D	W	R	E	K	2/12 (16.66)	3/12 (25.00)
*Zingiber officinale *	GP-II	**D**	D	L	**P**	D	**S**	I	D	W	R	E	N	2/12 (16.66)	3/12 (25.00)
*Pineapple stem *	Bromelain	—	A	V	**P**	Q	**S**	I	D	W	R	D	Y	1/12 (08.33)	2/12 (16.66)
Rat liver	Cathepsin J	**D**	T	**P**	A	**N**	E	T	Y	P	D	L	L	3/12 (25.00)	3/12 (25.00)

*Shared amino acids as bold.

**Table 4:** Comparison of N-terminal sequence of Nivulian-II with other matched fragments of known cysteine proteases.

Source	Type	Sequence*												Identities	Positives
*Euphorbia nivulia *	Cysteine	**D**	**F**	**P**	**P**	**N**	**T**	**C**	**C**	**C**	**I**	**C**	**C**	—	—
*Naegleria gruberi *	Cysteine	**D**	**F**	**P**	**P**	**N**	E	T	S	L	**G**	F	S	5/12 (41.66%)	6/12 (50.00%)
*Polysphondylium pallidum *	Cysteine	V	I	E	**P**	**N**	**T**	S	**C**	I	**I**	I	P	5/12 (41.66%)	5/12 (41.66%)
*Ricinus communis *	Cysteine	C	P	D	G	**N**	**T**	**C**	**C**	**C**	**I**	Y	E	6/12 (50.00%)	6/12 (50.00%)
*Hevea brasiliensis *	Cysteine	C	P	E	S	**N**	**T**	**C**	**C**	**C**	**I**	F	E	6/12 (50.00%)	6/12 (50.00%)
*Saccoglossus kowalevskii *	BP-10	G	P	A	**P**	**N**	**S**	**C**	**C**	I	**I**	A	**M**	5/12 (41.66%)	7/12 (58.33%)
*Mycoplasma hominis *	MAP	V	G	F	**P**	**N**	**T**	**C**	**C**	I	S	V	N	5/12 (41.66%)	5/12 (41.66%)

*Shared amino acids as bold.
