# Supplementary material for: Peptide Mass Fingerprinting and N-Terminal Amino Acid Sequencing of Glycosylated Cysteine Protease of Euphorbia nivulia Buch.-Ham
Source: J Amino Acids. 2013 Feb 17;2013:569527. doi: 10.1155/2013/569527 (PMC3588393; doi:10.1155/2013/569527)
Supplement: Supplementary file 1 — Figure S1: Prime peptide (1561.831) of Nivulian-II for MALDI-TOF/TOF fragmention analysis. Figure S2: MALDI-TOF/TOF fragment ion analysis of prime peptide of Nivulian-II (1561.831). [file 569527.f1.pdf]

SUPPLEMENTARY FIGURES

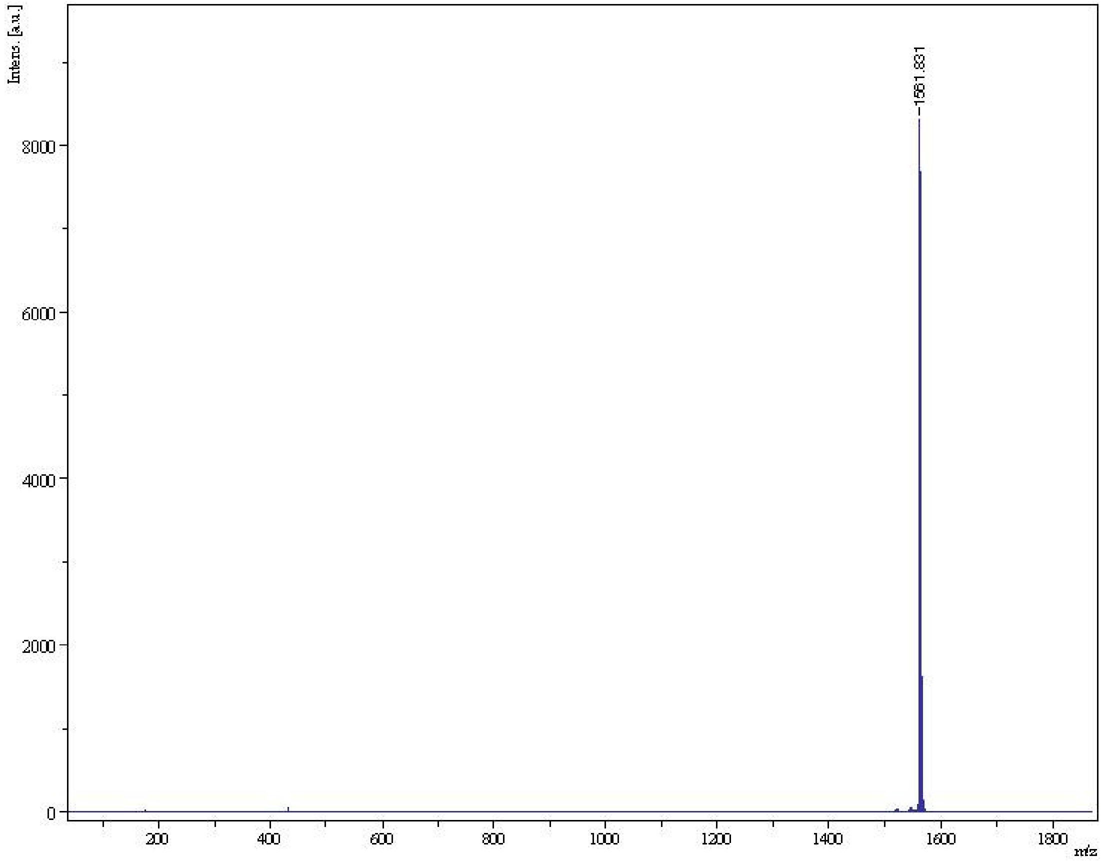

Figure S1. Prime peptide (1561.831) of Nivulian-II for MALDI-TOF/TOF fragment ion analysis.

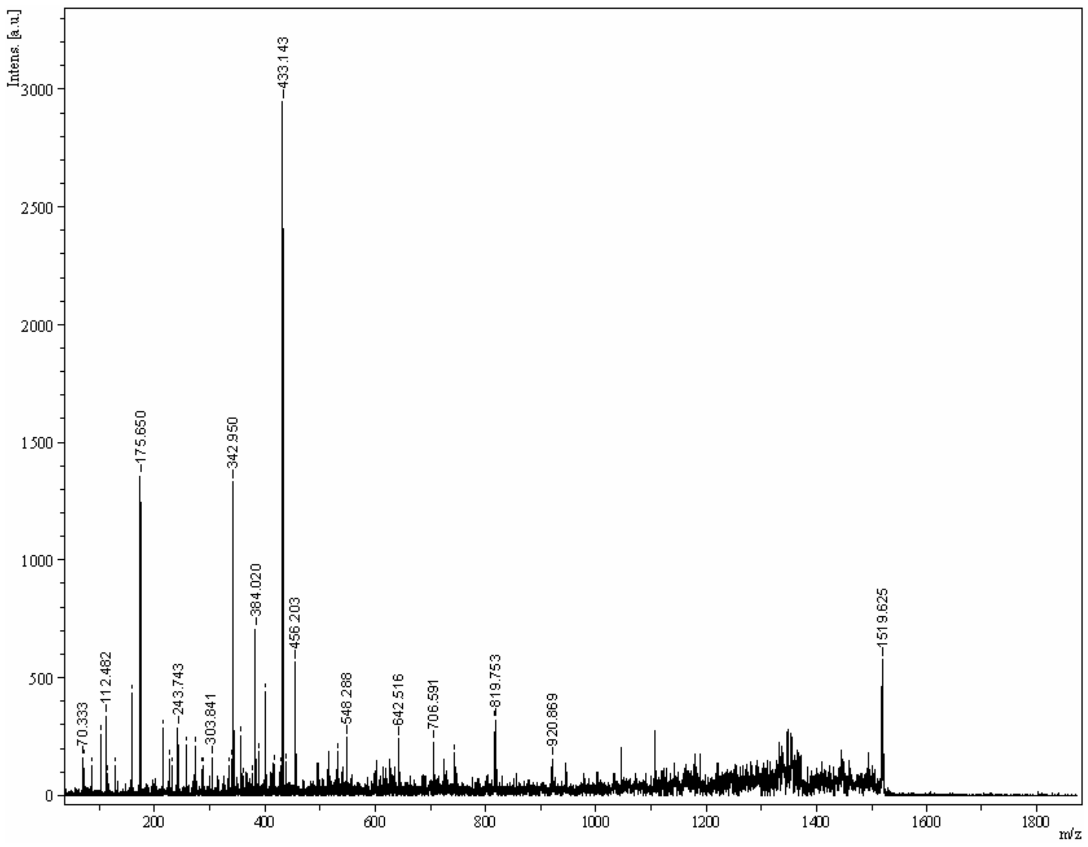

Figure S2. MALDI-TOF/TOF fragment ion analysis of prime peptide of Nivulian-II (1561.831).
